# Biochemical characterization of actinomycete from Namibia rocky crest mountainous soil and analyzing their bioactive metabolites for antagonistic effect against human respiratory pathogens

**DOI:** 10.11604/pamj.2024.48.12.33596

**Published:** 2024-05-14

**Authors:** Albertina Mariina Ndinelao Shatri

**Affiliations:** 1 Department of Human, Biological and Translational Medical Sciences, University of Namibia, Private Bag 13301, Mandume Ndemufayo Avenue, Pionierspark, Windhoek, Namibia

**Keywords:** Actinomycete, bioactive metabolites, *Streptococcus pneumonia*, *Stachybotrys chartarum*, resistance, antioxidants

## Abstract

**Introduction:**

the present study aimed at isolating and characterizing actinomycete from unexplored Windhoek rocky crest mountainous soil and extracting bioactive metabolites as possible therapeutics against common life-threatening Streptococcus pneumonia (S. pneumonia) and Stachybotrys chartarum (S. chartarum).

**Methods:**

chemotaxonomy and biochemical methods were used to identify the isolates. The solvent extraction method was used to extract bioactive compounds. Agar overlay and disc diffusion methods were used to determine the antimicrobial activity of isolates and extracted bioactive metabolites against S. pneumonia and S. chartarum. The antioxidant activity of the extracted bioactive metabolites was determined using 2.2-diphenyl-1-picrylhydrazyl (DPPH) free radical scavenging method with ascorbic acid as a positive control. Comparison between groups was done using a Two-way ANOVA, followed by Bonferroni post-test.

**Results:**

three distinct isolates from 3 soil samples were identified on starch casein agar and distinguished using biochemical tests. All three isolates showed strong inhibitory activity against S. pneumonia with average growth inhibition zones between 18.0±1.00 and 27±0.00 mm p< 0.005. All isolates showed potent inhibitory activity against S. chartarum with the average inhibition zones ranging between 42.0±1.00 and 48±0.00 mm, p< 0.005. The chloroform extracts showed potent DPPH activity of up to 73± 1.41%.

**Conclusion:**

growth conditions and extraction solvents can influence the antimicrobial and antioxidant properties of bioactive metabolites.

## Introduction

The discovery of streptomycin presented actinomycetes as a promising source of natural bioactive microbial metabolites to be used as next-generation medicine for different human conditions [1]. Given the biodiversity in the actinomycetes genera, there is hope for future actinomycete-derived medicine, especially for respiratory complications [2]. During the idio-phase, actinomycetes produce low molecular mass products called secondary metabolites or bioactive compounds. Bioactive metabolites are not essential for the growth of the bacteria that produce them, but are essential for diverse survival functions in nature [3]. When culturing actinobacteria, a balance between the right content of carbon source and other nutrients is significant to produce bioactive metabolite with antagonistic effects [4]. There is an increase in health complications caused by resistant respiratory bacteria, viruses, and fungi which calls for an agent need to screen for next-generation respiratory medicine [5]. Exposure to fungus such as *Stachybotrys chartarum* is linked to serious complications such as muscle aches, headaches, cough, pulmonary hemorrhage, dermatitis, and interstitial lung disease [6]. *S. chartarum* produces mycotoxins, such as macrocyclic trichothecenes, which inhibit protein and DNA synthesis, induce protein degradation, disrupt cellular function, and cause cellular injury and inflammation. *S. chartarum* also releases stachylysin which can lead to pulmonary hemorrhage [7].

Studies have reported irrational uses of antimicrobials to treat conditions arising due to exposure to fungus including *S. chartarum*, which have resulted in higher cases of resistance to the available antifungal treatments [8]. *S. pneumonia* is associated with a higher mortality rate of 1.6 million globally, with most cases reported in African countries [9]. *S. pneumonia* is alpha-hemolytic; hence it can break down red blood cells through the production of hydrogen peroxide which can also cause DNA damage, and kill cells within the lungs [10]. *S. pneumonia* also causes fever and chills, coughs, difficulty breathing, and chest pain. In severe cases, once *S. pneumonia* has spread to the brain and spinal cord, it can cause pneumococcal meningitis [10]. *S. pneumonia* is also responsible for sinusitis, septic arthritis, osteomyelitis, peritonitis, and endocarditis cases [11]. The daily reports on multidrug resistance among these respiratory pathogens have prompted a need to discover next-generation medicine [12]. Studies conducted globally have reported that 90 % of bioactive medicine is derived from Actinobacteria [12]. Therefore, the main objective of the present study was to isolate and characterize Actinomycete isolated from Windhoek rocky crest Mountain soil and to screen their novel bioactive metabolites as possible therapeutic for life in the threatening *S. pneumonia* and *S. chartarum* pathogens.

## Methods

**Study area:** the sampling site in this study was rocky crest mountainous area, Windhoek, Namibia and sampling was done in December 2020. This is an unexplored area with a GPS coordinate of 22°34'15.2"S 17°02'45.0"E. This is a dry mountainous area with undisturbed vegetation and an average rainfall of approximately 285 mm and an average temperature of 18.5°C per year [13].

**Figure 1:**
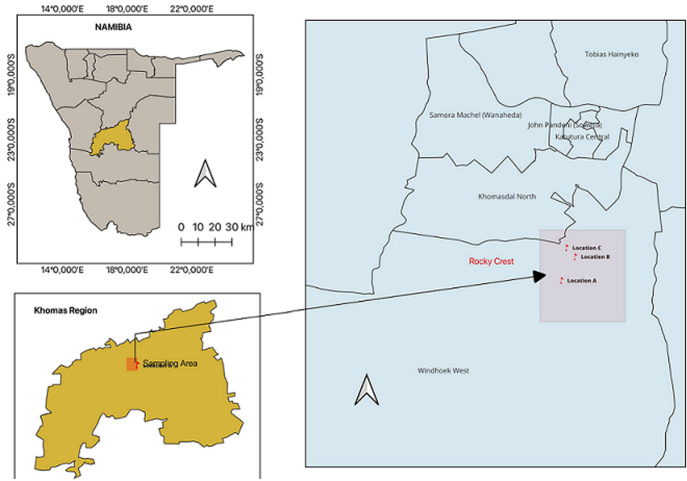
the map of the rocky crest mountainous study area designed by Haindongo, in 2023

**Sampling:** samples were randomly collected from 3 different sites around the unexplored rocky crest mountainous area as depicted in the map in Figure 1 that was designed by Haindongo, 2023 for this study. From each site, 5 soil samples were collected and mixed to represent 1 sample of each location. The soil samples were collected at a 9cm upper depth layer of soil into sterile plastic bags and immediately transported to the Microbiology laboratory at the Hage Geingob Campus.

**Sample processing and isolation of actinomycete:** in the microbiology laboratory at the Hage Geingob Campus, the samples were dried by heating at 100°C for 2 hours, and treated with 1.5% phenol for 25 minutes at 30°C. Serial 10-fold dilutions of the suspension were prepared and 0.1 ml of 10-2, 10-3, and 10-4 dilutions were spread on the surface of starch casein nitrate agar containing (starch 0.1%, sodium caseinate 0.03%, KNO_3_ 0.2%, and agar 1.5%, pH 7.1). Each agar plate was supplemented with antibiotics, 50 mg/L of cycloheximide, and 20 mg/L of nalidixic acid. The plates were incubated at 37°C for 14 days and different colonies were picked and sub-cultured on starch casein agar. The pure isolates were observed for their cultural characteristics by microscopic and macroscopic methods. All pure isolates were preserved in 80% (v/v) glycerol solution at -80°C until further use [3].

**Morphological characterization of isolates:** all pure isolates were inoculated on starch casein agar and incubated for 14 days at room temperature. Cellular morphologies were observed under the light microscope at X40 and characteristics such as color, aerial and substrate mycelium were noted.

**Chemotaxonomy characterization of isolates:** cell wall composition was analyzed according to the method of Red Cross Climate Centre (2021) [14], with minor modifications. The culture was grown in starch casein broth for 10 days. After the incubation period, the mycelia were separated by centrifugation at 5,000 rpm for 10 minutes and washed three times with deionized water. The cells were then examined for the composition of amino acids and sugars.

**Identification of amino acids:** the cell wall samples of 0.3 ml were titrated with hydrochloric acid (HCL) to give a final concentration of 6N HCl at 100°C for 15 hours. The hydrolyzed materials were transferred to 100 ml beakers and dried overnight. Using a capillary tube, a few drops of the same sample were spotted on thin-layer chromatography and run using n-butanol, acetic acid, and water (4: 1: 1) as a solvent system. The presence of amino acids was identified by spraying with 0.25% Nihydrin and drying at 100°C in the oven for 4 minutes.

**Identification of sugars:** the cell wall sample of 1 ml was taken and HCL was titrated on it to give a final concentration of 2N HCl in a sealed glass bottle. The bottle was placed in a boiling water bath for 1 hour set at 100°C. The hydrolyzed materials were transferred to 100 ml beakers and dried overnight. To this, water was again added and the process was repeated three times. The materials were finally suspended in 700µl of water. Then using a pipette, 100µ l of the sample was spotted on a thin layer of chromatography paper, and n-butanol, acetic acid, and water (3: 1: 1) mixture was used as a solvent system. The chromatogram was then sprayed with a solution containing 0.4g silver nitrate, 0.9 ml water, and 22 ml of 95% ethanol as a chromogenic reagent. The chromatogram was finally dried until the appearance of dark spots [15].

**Physiological and biochemical characterization of the selected isolate:** the growth of to isolate was tested at different temperatures between 20 and 40°C, pH ranges of pH 5.0 to 10.0, and different concentrations of NaCl (2-10%w/v). The carbohydrate utilization was tested on various carbon sources such as galactose, glucose, fructose, sucrose, xylose, and meso-inositol. Biochemical tests such as fermentation of carbohydrates, starch hydrolysis, nitrate reduction, casein hydrolysis, citrate utilization tests, indole, oxidase, catalase, and urea, were also carried out for all the samples [16].

**Inoculum of pathogenic organisms:** all the actinomycetes were tested for antibacterial activity and antifungal activity. Antibacterial activity against *S. pneumonia* ATCC 27336 and antifungal activity against *S. chartarum*. The bacterial cultures were sub-cultured on nutrient agar medium and incubated at 37°C for 24 hours. Fungal cultures were sub-cultured on sabouraud dextrose agar medium at 25°C for 48 hours. The turbidity of the pathogenic organisms was adjusted to be equivalent to 0.5 McFarland standard using nutrient broth (1.0 x 10^7^ CFU/ml). The experiments were conducted in triplicates. The pathogens were kindly supplied by the Department of Microbiology, Faculty of Health Science, Hage Geingob Campus.

**Antibacterial and antifungal activity of pure actinomycete isolates:** the antagonistic activity was determined by the agar overlay method. Actinomycete isolates were spot inoculated on starch case in agar plates and incubated at 37°C for 10 days. Subsequently, the plates were overlaid with nutrient agar and Sabouraud dextrose agar medium with 0.0075 g/ml concentration of agar seeded with *S. pneumonia* or *S. chartarum* cultures respectively. The plates with *S. chartarum* were incubated at 25°C for 48 hours and the plates with *S. pneumonia* were incubated at 37°C for 24 hours. The diameters of the inhibition zone formed around the colonies were recorded in millimeters after incubation [17].

**Fermentation and purification of bioactive metabolites:** the production of the bioactive metabolite was carried out by submerged fermentation using a starch casein production broth. Starch casein broth was used as an inoculum medium to increase the cell mass of to isolate. Pure colonies of the isolates were cultivated in 50 ml of the sterilized Starch casein broth medium and kept on a rotary shaker set at 150 rpm at 37°C for 4 days. After 4 days, 20 ml of the metabolically active inoculum were transferred into 500 ml of the production medium in 1000 ml Erlenmeyer flasks. The flasks were incubated for 14 days at 37°C and 150 rpm on a Juglba SW 22 Shaker. The cultures were later centrifuged at 10,000 rpm for 35 minutes. The cell debris and other cell components were discarded, while the cell-free supernatant was extracted with equal volumes of chloroform or n-hexane. The combined chloroform or n-hexane fractions were then evaporated by rotary evaporation to obtain the extracts that were used for secondary antimicrobial screening. The disk diffusion method on the Whatman filter paper with a 6 mm diameter was used to determine the antimicrobial activity of the extracts against the test bacteria on Mueller Hinton agar plates [17].

**Antibacterial activity of extracted bioactive metabolites:** the densities of the *S. pneumonia* and *S. chartarum* suspensions were determined by diluting the broth culture to 1: 100. This was achieved by mixing 0.1 ml of the inoculum and 9.9 ml of nutrient broth. The dilutions were compared with 0.5 McFarland standards (1.0x10^7^ CFU/ml). Using a sterile rod, 0.1 ml of *S. pneumonia* and *S. chartarum* respectively was inoculated and spread evenly onto the sterile Muller Hinton agar plates. The 6mm filter discs were sterilized in the autoclave and were soaked in 100 l of the secondary metabolites (extracts). All the experiments were performed in triplicates and plates were immediately incubated at 37°C for 24 hours. Each zone of inhibition was measured with a ruler in mm and the mean was calculated [18]. Chloroform and n-hexane were used as negative controls while ampicillin and ceftriaxone were used as positive controls.

**Antioxidant activity:** the 2.2-diphenyl-1-picrylhydrazyl (DPPH) radical scavenging activity of the chloroform and n-hexane secondary metabolites at 125 µg/ml was determined. The concentration was chosen as it is the average MIC for both *S. pneumonia* and *S. chartarum*. About 2 ml of 0.002% DPPH solution was added to each tube with the secondary metabolites, mixed, and incubated for 30 minutes in the dark. The reduction of DPPH radical was quantified at 517 nm using a UV-V is spectrophotometer [19]. The percentage of DPPH radical scavenging activity was calculated as: DPPH radical scavenging activity %= [(Ac - As)/Ac] x100. Where, Ac and As were the absorbance of the control and sample, respectively. The experiment was repeated three times.

**Statistical analysis:** all experiments were done in triplicates and statistical analysis was performed employing Graph Pad Prisms software version 7.0. Comparison between groups was done using two-way ANOVA, followed by Bonferroni post-tests. The comparisons were made between the antibacterial and antifungal activities respectively of different secondary metabolites and isolates against *S. chartarum* and against *S. pneumonia*. All data were presented as mean ± Standard deviation. Results for total phenol quantification, antioxidant activity, and antibacterial activity were considered to be statistically significant P<0.005.

## Results

**Figure 2:**
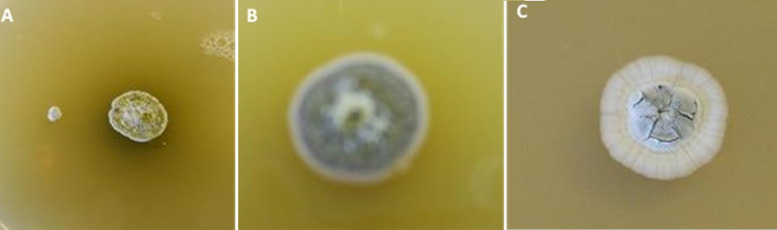
colonies morphological characteristics of actinomycete isolates: A) AS-1; B) AS-2; C) AS-3 on starch casein agar

**Figure 3:**
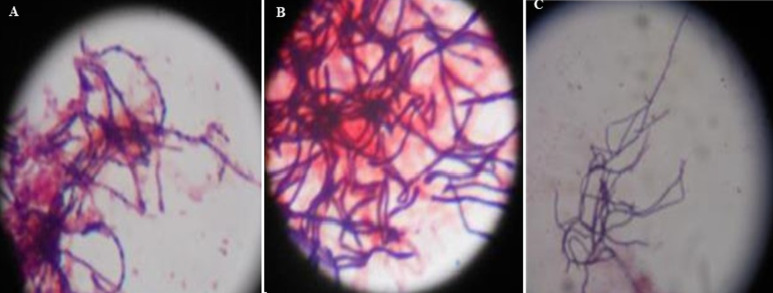
microscopic morphological characteristics of aerial hyphae and spore chain structure of: A) AS-1; B) AS-2; C) AS-3 (light microscopy X40)

**Isolation and characterization of the isolates:** Table 1 shows the 3 distinctive isolates collected from the rocky crest mountainous soil. Actinomycetes were cultured on starch casein agar. The isolated strain showed different identification characteristics. The isolated actinomycetes required oxygen for growth and were nonmotile. Isolate 1 (AS-1) was chalky white, isolate 2 (AS-2) was gray, and Isolate 3 (AS-3) was white with a scaly shell (Figure 2). The isolates grew rapidly on starch casein agar and had an earthy odor. The aerial and substrate mycelium of different colors were formed by the isolates (Table 1). The sizes of the pure colonies were ≤ 7cm. The thin and short mycelia were observed under light microscopy X40 (Figure 3). The chemotaxonomic analysis showed the presence of LL-diaminopimelic acid in the peptidoglycan cell walls of isolate AS-1, AS-2, and AS-3 (Table 1). Isolate 1 showed the ability to utilize all different carbon sources for cellular respiration. However, isolating AS-2 and AS-3 could not utilize galactose, xylose, and meso-inositol. Although all isolates grew at temperatures between 25 and 40°C, none of the isolates grew at 4°C. All isolates grew between the pH of 5 and 10 and were tolerant to 7% sodium chloride (Table 1).

**Table 1:** growth, use of carbon, tolerance of pH, temperature, and sodium chloride, physiological and biochemical characteristics of different actinomycete isolates

Tests	AS-1	AS-2	AS-3
**Growth**			
Substrate mycelium	White	Gray	White
Soluble pigment color	Clear	Brown	Green
Aerial mycelium	White	Brow	White
**Carbon use**			
Fructose	P	P	P
Galactose	P	A	A
Mannose	P	P	A
Sucrose	P	P	P
Meso-inositol	P	A	A
Xylose	P	A	A
Glucose	P	P	P
**pH tolerance (5-10)**	Yes	Yes	Yes
**Temperature**			
Growth at 4°C	No	No	No
Growth at 25- 40 °C	Yes	Yes	Yes
**Sodium chloride tolerance**			
Sodium chloride below 2%	no	No	no
Sodium chloride at 10%	yes	yes	yes
**Physiological and biochemical characteristics of isolates**			
Tests	AS-1	AS-2	AS-3
Gram staining	Yes	Yes	Yes
Starch hydrolysis	Yes	Yes	Yes
Casein hydrolysis	Yes	Yes	Yes
Citrate utilization	Yes	Yes	Yes
Methyl red	No	Yes	Yes
Indole production	Yes	Yes	Yes
Nitrate reduction	No	No	Yes
Catalase	Yes	Yes	Yes
Urea	Yes	Yes	Yes
Oxidase utilization	No	Yes	No
Presence of LL-DAP in Cell wall composition	Yes	Yes	Yes
Presence of meso-DAP in Cell wall composition	No	No	No

P: present; A: absent

**Figure 4:**
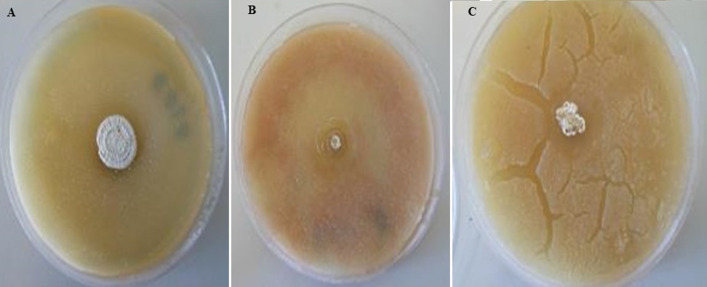
initial screening of antibacterial activity by Agar overlay method for: A) isolate 1 (AS-1); B) isolate 2 (AS-2); C) isolate 3 (AS-3)

**Antagonistic activity of the isolates on solid media with *S. pneumonia* and *S. chartarum*:** all three isolates showed strong inhibitory activity against *S. pneumonia* with average growth between 18.0±1.00 and 27±0.00 mm. All isolates showed very strong antifungal activity against *S. chartarum* with the inhibition zone ranging between 42.0±1.00 and 48±0.00 mm (Table 2), examples of inhibition zones are shown in (Figure 4A, B, C).

**Table 2:** antagonistic activity of the isolates using the perpendicular streak method

Isolates	Antibacterial activity (mm)	Antifungal activity (mm)
**AS-1**	18.0±1.00*	42.0±1.00*
**AS-2**	22.5±2.00*	45.5±2.00*
**AS-3**	27±0.00*	48±0.00*

Average inhibition: ± Standard deviation; n=3; *: p< 0.0001 significance difference between antibacterial and antifungal activity

**Antimicrobial activity of extracted bioactive metabolites:** antimicrobial properties of the bioactive metabolites extracted with different organic solvents were determined by measuring the inhibition zone around a Whatman paper disc with the bioactive metabolite. The difference in polarity index between chloroform (4.1) and n-hexane (5.1) could have contributed to the differences in the antibacterial and antifungal activity of chloroform and n-hexane extracts reported in this study. Among all extracts, AS-3 showed the lowest MIC values of chloroform and n-hexane extracts (100 µg/ml), against *S. pneumonia* and *S. chartarum* respectively. It is interesting to note that the bioactive secondary metabolites extracted in this study showed strong inhibitory activity against ampicillin-resistant *S. pneumonia* when compared to ceftriaxone (Table 3).

**Table 3:** antimicrobial activity of chloroform and n-hexane extracts produced from actinomycetes

Average inhibition zone diameter (mm) for chloroform extracts			MIC (µg/ml) for chloroform extracts		Average inhibition zone diameter (mm) for n-hexane extracts		MIC (µg/ml) for n-hexane extracts	
**Isolates**	***S. pneumonia***	***S. chartarum***	***S. pneumonia***	***S. chartarum***	***S. pneumonia***	***S. chartarum***	***S. pneumonia***	***S. chartarum***
AS-1	13.0± 1.00*	30.0±1.00*	250	125	0.0±0.00	0.0±0.00	ND	ND
AS-2	18.5±2.00*	36.5± 2.00*	125	250	0.0±0.00	0.0±0.00	ND	ND
AS-3	22.0±0.60*	37.0±0.00*	100	250	8.0±0.60	11.0±0.00	125	100
Chloroform					0.0±0.00	0.0±0.00	ND	ND
n-hexane					0.0±0.00	0.0±0.00	ND	ND
Ceftriaxone					20.0±0.60	ND	ND	ND
Ampicillin					0.00±0.00	ND	ND	ND

Average inhibition ± SD; n=3; *: p< 0.0001 between antibacterial and antifungal activity, 0: No activity, ND: not determined

**Figure 5:**
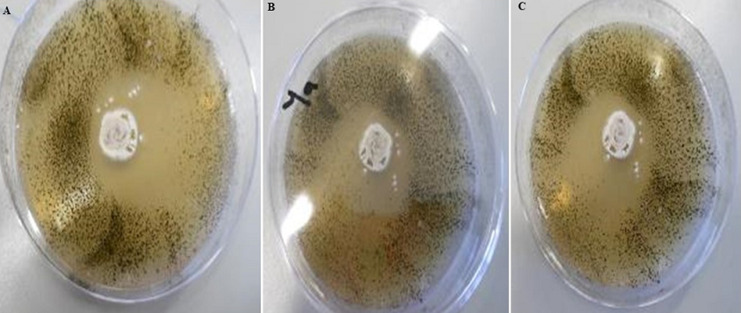
initial screening of antifungal activity by Agar overlay method for: A) AS-1; B) AS-2; C) AS-3

**Antioxidant activity of secondary metabolites:** the results of DPPH radical scavenging activity of the chloroform and n-hexane bioactive metabolites extracted from isolates 1-3 are depicted in (Figure 5). The chloroform extracts showed a DPPH free radical scavenging activity between 66±0.82 and 73± 1.41%; while the n-hexane extracts showed DPPH free radical scavenging activity between 59.33± 0.94 and 65. 3± 1.25% as compared to ascorbic acid which showed 80.6%± 0.47 activity.

**Figure 6:**
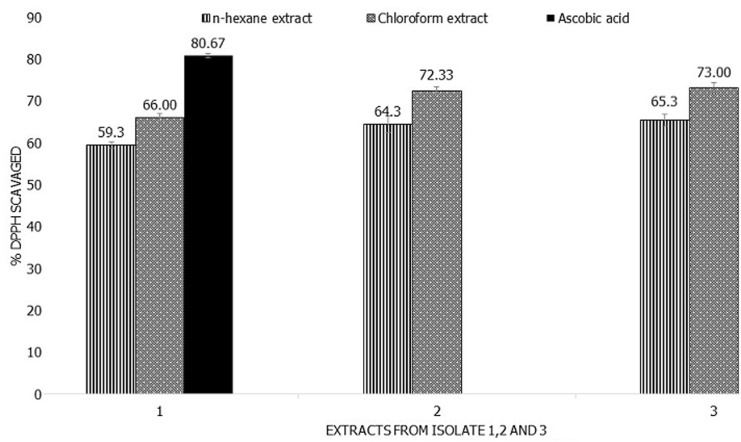
antioxidant activity of chloroform and n-hexane secondary metabolites; data are average ± SD values for 3 independent repeated experiments; 1-3 represent bioactive metabolites from isolates 1-3; DPPH: diphenyl-1-picrylhydrazyl

## Discussion

Actinomycetes are reported to possess antagonistic activity and therapeutic properties against pathogenic bacteria due to their ability to produce secondary metabolites [20]. With increases in respiratory diseases caused by *S. pneumonia* and *S. chartarum*, it is of utmost interest to research unexplored land for actinomycetes with antibiotic-producing abilities against human life-threatening *S. pneumonia* and *S. chartarum* respiratory pathogens. When culturing actinomycetes it is important to first inhibit the growth of other fast-growing bacterial colonies that may be present in the soil samples. Hence pretreatment of the soil suspension by using 1.5% phenol is mandatory as it allows the recovery of less-abundant phenol-resistant actinomycetes while eliminating other bacteria, and fungi, by denaturing their proteins or by disrupting their cell membrane [21]. The presence of LL-diaminopimelic acid showed that the isolates are *Streptomyces* and had no *meso*- diaminopimelic acid, which is mostly reported in pathogenic actinomycetes [22]. In this study, the isolated actinomycetes have shown positive results for indole and catalase tests, and they can grow on different carbon sources. This indicates that the isolates can decompose the amino acid tryptophane to indole. Literature has shown that the genus of Nocardia is catalase-positive and can hydrolyze several types of sugars. However, the genus Jonesia is catalase-positive with oxidase-negative, does not hydrolyze indole, hydrolyzes urea, and citrate, liquefies, and ferments sugars such as glucose, lactose, and saccharose [23].

The increasing microbial resistance and the limited availability of antibacterial and antifungal complications present a great need for the development of a novel class of therapeutic compounds with minimal side effects [10,24]. *S. pneumonia* and *S. chartarum* infections have claimed numerous lives in the past decades. Hence, there is a need to find alternative treatments for these pathogens. The screening of the antibacterial and antifungal activity of actinomycete isolates in this study shows potent antagonistic properties in comparison with pure liquid bioactive compounds. Studies have linked this to the fact that the cultivation of solid and liquid media may lead to the production of different active antibiotics and some compounds may be lost during the organic solvent extraction, because the active components may become inactivated during the extraction step as observed for n-hexane and chloroform extracts [25]. Studies have been conducted globally evaluating the antibacterial activity of actinomycetes against *Escherichia coli, Staphylococcus aureus* strains A and B, *Bacillus species* strain *A and B*., *K. pneumonia, S. viridians, Pseudomonas, Klebsiella*, and *Pseudomonas aeruginosa* [26,27]. However, the efficacy of actinomycete against *S. pneumonia* and *S. chartarum* has not been documented, and this makes the findings of this report first in documenting the antagonistic activity of bioactive compounds from actinomycetes against *S. pneumonia* and *S. chartarum* the first. The sizes of the inhibition zones for chloroform crude extracts showed that the isolates have a significant effect in eliminating *S. pneumonia* and *S. chartarum*.

While numerous antibiotics have been discovered from actinomycetes, these only signify a small fraction of the selection of existing bioactive compounds produced. Therefore, extraction and characterization of promising bioactive compounds from actinomycetes is a valuable endeavor [27]. In recent years, researchers have focused on the analysis of natural antioxidant and their health benefits. According to Dholakiya *et al*. 2017 [19], actinobacteria isolated from the Gulf of Khambhat, Gujarat have shown antioxidant activity of up to 82.86%. Another study by Mohammadipana *et al*. 2018 [28] reported 26.8% antioxidant activity of the isolated secondary metabolites. These findings agree with the antioxidant findings of this study since all three extracts from isolates 1, 2, and 3 in this study showed antioxidant activity at 59.3%. This shows that secondary metabolites from actinomycetes can inhibit oxidation. The higher antibacterial activity and antioxidant activity of chloroform-extracted bioactive metabolites in this study could be due to the higher antioxidant activity present in these extracts as compared to n-hexane extracts. Studies have proven that antioxidants can prevent the formation of free radicals differently through cellular mechanisms. The antioxidant potential of actinomycetes allows the prevention of chain initiation, decomposition of peroxides, a transition of metal ion catalysts, and hydrogen abstraction [28,29].

## Conclusion

Actinomycetes isolated in this study are promising sources of next-generation antimicrobial for resistant respiratory pathogens such as *S. pneumonia* and *S. chartarum*. This is the first report to show the broad-spectrum antibacterial and antifungal potential, as well as the higher presence of antioxidants in the bioactive metabolites extracted from the unexplored rocky Crest mountainous soil. This could be an important source for the exploration of next-generation bioactive metabolites for treating human health complications caused by *S. pneumonia* and *S. chartarum*. Future studies should also consider sampling at different seasons to evaluate the effect of seasonal changes on the quality of secondary metabolites, as well as to identify the secondary metabolites by high throughput technics such as Higher Performance Liquid Chromatography.

### What is known about this topic

- *Most of the currently known antimicrobials were originally isolated from actinomycetes;*
- *There is a higher resistance pattern to the available antibiotics used to treat upper and lower respiratory infections;*
- *The search for safe and effective novel next-generation bioactive metabolites for treating respiratory ailments is momentous*.

### What this study adds

- *The isolation of actinomycetes from an unexplored ecosystem is one way of discovering a broad spectrum of next-generation antimicrobial agents;*
- *The fermentation and extraction solvent can influence the antimicrobial and antioxidant properties of the bioactive metabolites*.
